# Corneal Remodeling Using Laser Asymmetric Keratectomy in Patients with Keratoconus Suspect

**DOI:** 10.3390/jcm14186568

**Published:** 2025-09-18

**Authors:** Byung Moo Min, Hee Jong Cheon

**Affiliations:** 1 Woori Eye Clinic, Department of Ophthalmology, Yonsei University College of Medicine, Daeduk Daero 219, Dunsan-dong, Seo-gu, Daejon 35229, Republic of Korea; 2 GJ St. Mary Eye Clinic, Wongjin-Ro 223, Gongju 32539, Republic of Korea; tongyng@gmail.com

**Keywords:** corneal remodeling, KCS, focal corneal steepening, asymmetric peripheral corneal thickness, L-LAK, corneal symmetry

## Abstract

**Background:** The aim of this study was to assess a corneal remodeling technique using laser asymmetric keratectomy (C-LAK) treatment, and its outcome for keratoconus suspect (KCS) by evaluating corneal regularity. **Methods:** In this retrospective case–control study, 34 eyes of 20 patients with KCS were studied before and 1 year after L-LAK. This new technique could ablate the original refractive errors, the thicker peripheral cornea, and myopia induced by LAK simultaneously (crescentic customized ablation). Before and 1 year after the operation, the refraction, UDVA, keratometry, and corneal symmetry evaluated as the total corneal central-thickness deviation (SUM) and the distance between the maximum posterior elevation (best-fit-sphere [BFS]) and the visual axis (DISTANCE) were compared. **Results:** Twenty patients with KCS aged 41.0 ± 13.5 years were evaluated. Preoperatively, the spherical equivalent (SE, D) −2.57 ± 1.64 and the Kmax was +48.21 ± 0.89 D. At 1 year postoperation, 79.4% (27/34) of the eyes had a UDVA of 20/20 or better. The SE and Kmax (D) were −0.40 ± 0.43, 44.47 ± 1.38 (Ps = 0.001, respectively), the corneal symmetry was better due to the decrease in the SUM (µm) (from 141.88 ± 48.24 to 66.21 ± 15.22) and DISTANCE (mm) (from 1.11 ± 1.14 to 0.46 ± 0.40). No postoperative corneal ectasia appeared. **Conclusions:** L-LAK made the corneas symmetric by decreasing the SUM and DISTANCE, decreased focal steepening, and showed good 1-year postoperative outcomes.

## 1. Introduction

In order to avoid postoperative corneal refractive surgery complications, it is very important to detect keratoconus (KC) in its earlier stage, including keratoconus suspect (KCS) [1,2,3,4,5,6]. KCS shows several characteristics such as a steep focal keratometric curvature more than 47.0 D, peripheral corneal asymmetry, etc., but does not result in worse visual impairment compared to keratoconus (KC) [1,2,3,4,5,6]. Until now, a definition of KCS has not been determined [5,6,7,8,9]. Currently, corneal Scheimpflug shaping and epithelial evaluation by anterior OCT are utilized [10,11,12]. Of the suspected findings of KCS, a corneal focal curvature greater than +47.0 D and SUM ≥ 80 µm could be the most important for determining whether laser refractive surgery should be performed in myopic patients with KCS [13,14,15,16,17,18] because of the absolute contraindication to laser refractive surgery due to complications after operation [19,20,21,22,23,24,25,26,27,28,29], possibly because of biomechanical effects causing optical aberrations [22,23,24,25,26,27,28,29].

Recently, asymmetric corneal ablation methods have been developed to attempt to make the asymmetric cornea found in KC, corneal ectasia, and KCS symmetric postoperatively with selective ablation of the thicker portion of the cornea [30,31,32,33]. In KC, a novel approach to crescent keratectomy with an excimer laser and a mask resulted in good visual acuity, keratometry, and corneal morphology at 1 year postoperation [30]. In corneal ectasia, keratectomy with the edges of the resection sutured led to corneal flattening at 3 year postoperation [31]. Also, in patients with KCS, quantitative asymmetric corneal crescentic ablation on the thicker peripheral cornea, customized with LAK, and simultaneous ablation of the original refractive errors to make the cornea symmetric and to correct the refractive errors postoperatively could be safe and good outcomes have been shown without postoperative ectasia and focal corneal steepening [32,33]. Postoperative corneal symmetry is an important factor in clinical outcomes. The DISTANCE and SUM according to an Orbscan map as evaluating indices are very useful [13,14,15,16,17,18].

LAK as a customization method has been reported to lead to excellent clinical outcomes with symmetric corneas in patients with peripheral asymmetric corneas [13,14,15,16,17,18,32,33].

C-LAK could simultaneously correct the original refractive errors and make the cornea symmetric by decreasing the SUM and DISTANCE with customization ablation (crescentic customized ablation), decreasing the steep keratometric curvature of the cornea (Kmax) compared with conventional laser refractive surgery such as LASIK, etc. [13,14,15,16,17,18,32,33], and could show good postoperative corneal symmetry and visual outcomes without postoperative complications in patients with KCS [32,33].

The purpose of our study was to report the change in corneal symmetry and clinical outcomes after L-LAK in patients with KCS manifesting as a SUM ≥ 80 µm and corneal focal curvature greater than +47.0 D on the thinner corneal portion by evaluating the corneal regularity in order to prevent postoperative complications, which is one of the leading challenges in laser refractive surgery without LAK.

## 2. Materials and Methods

This study included 34 eyes of 20 patients who received L-LAK at the Woori Eye Clinic between March 2021 and December 2021. KCS was diagnosed the same as in our previous studies, using the criterion of a corneal focal curvature greater than 47.0 D [1,2,3,4,5,6,7,8,9].

The inclusion criteria were patients with KCS manifesting as both a corneal focal curvature greater than 47.0 D on the thinner corneal portion and a SUM of ≥80 µm [13,14,15,16,17,18], those that underwent an L-LAK operation, those manifesting myopia > −1.50 diopter and 20/20 or better in CDVA, and those whp received a 1-year postoperative follow-up check. Patients that underwent a follow-up examination less than 1 year postoperation, and those with a history of other ocular operations, corneal scarring, pregnancy, glaucoma, and causes of ocular astigmatism were excluded.

**Figure 1:**
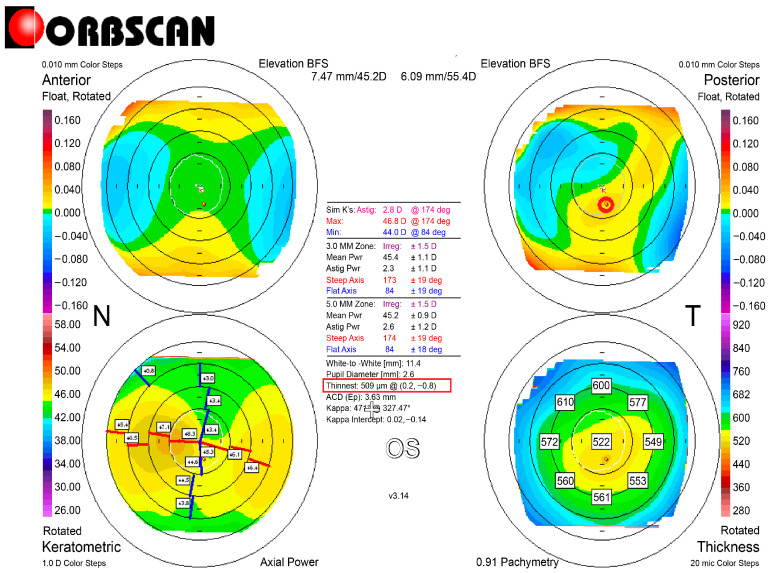
Pre-C-LAK Orbscan map. Right bottom shows a pachymetric map of an example of SUM: 136 µm. Right top map: Right top map shows Kmax is 48.3 D. The red box and circle shows the thinnest point.

**Figure 2:**
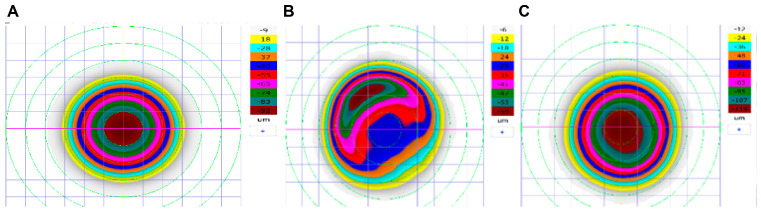
(**A**) Original ablation plan of refractive errors (−7.50 D) of case. (**B**) Customization ablation plan. (**C**) C-LAK ablation plan (total ablation = original ablation + crescentic customized ablation).

**Figure 3:**
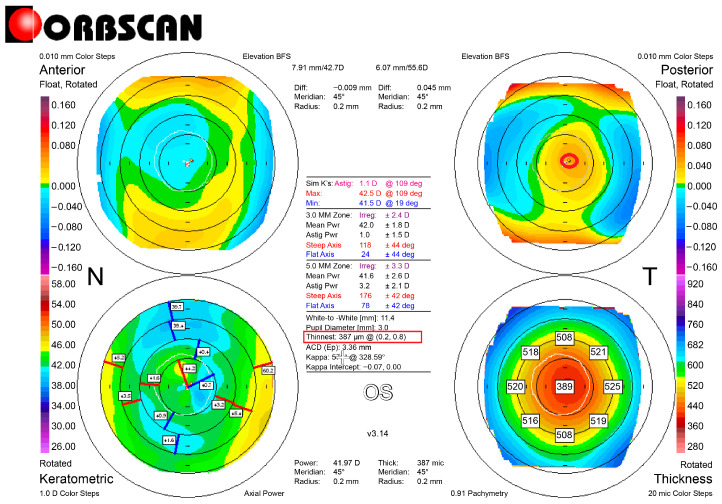
Post C-LAK Orbscan map. Right bottom map shows SUM: 11 µm. Right top map shows Kmax is measured as 44.2 D.

**Figure 4:**
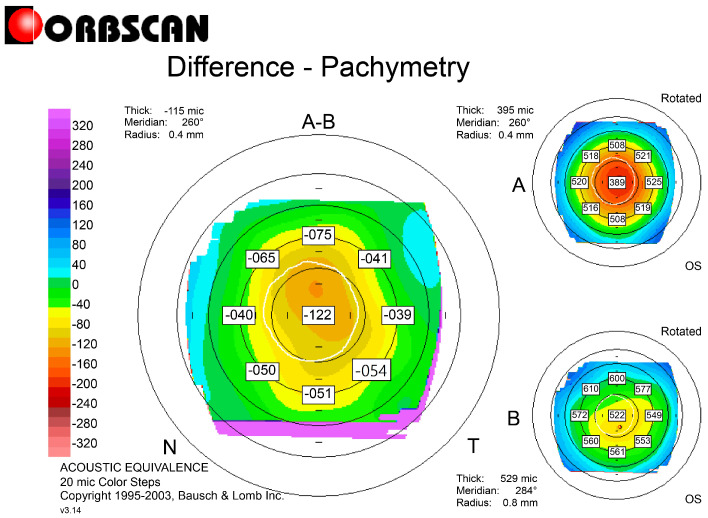
Differential pachymetric map between pre- and 1-year post-C-LAK.

Refractive errors were corrected with L-LAK using an excimer laser (Kera Havest Inc., Chiayi City, Taiwan) by the same doctor (BM Min) and with the same method (L-LAK) under topical anesthesia (Alcaine, Alcon NV, Vilvoorde, Belgium) for all patients [13,14,15,16,17,18,32,33]. The optic zone and the 7–9 mm transitional zone were the same as in previous studies (Figure 1) [13,14,15,16,17,18,32,33]. We operated using original ablation (Figure 2A) and crescentic customized ablation (Figure 2B). Therefore, we were able to correct both refractive errors and corneal morphology to create a symmetric cornea (total ablation) (Figure 2C Figure 3 and Figure 4) [13,14,15,16,17,18].

At 1 year pre- and postoperation, the following variables were analyzed: UDVA measured at a distance of 6 m, CDVA, spherical equivalent, keratometry, intraocular pressure, central pachymetry, pupil size, SUM, DISTANCE, kappa angle, TBUT (second), and tear osmolarity.

For statistical analyses, the paired t test was used in the case of variables following normal distribution and Wilcoxon signed rank test was used in the case of variables not following normal distribution. A *p* value of less than 0.05 was considered to be statistically significant. The data analysis was performed using IBM SPSS software version 25. Refractive outcomes were assessed using standard graphs and terms for refractive surgery results, and data analysis was performed using Microsoft Excel and R software version 4.2.3.

## 3. Results

The average age of the twenty patients was 41.0 ± 13.5 years (7 males and 13 females). Fourteen patients received the operation on both eyes and six received the operation on one eye. The average follow-up period (months) was 24.1 ± 2.3 (Table 1). For laser ablation, the optic zone (mm) for C-LAK was 6.35 ± 0.09, the ablation depth (µm) of the central cornea was 64.53 ± 24.41 for L-LAK, the induced myopia (diopters) due to LAK was −1.92 ± 0.48, and the residual stromal depth (µm) was 444.26 ± 36.20 with L-LAK (Table 2).

According to the preoperative to 1-year postoperative findings, the SE (diopters) ranged from −2.57 ± 1.64 to −0.40 ± 0.43 (*p* = 0.001); the sphere values (diopters) ranged from −2.12 ± 4.31 to −0.21 ± 0.43 (*p* = 0.001); and the cylinder values (diopters) ranged from −1.02 ± 2.27 to −0.45 ± 0.42 (*p* = 0.003). There were no astigmatic changes. The IOPs and pupil sizes were nearly same before and 1 year after the operation (Ps > 0.05) (Table 3). The Kmean (diopters) and focal steep keratometric curvature (Kmax) decreased from +45.23 ± 0.93 to +42.67 ± 1.84, and from +48.21 ± 0.89 to +44.47 ± 1.38, respectively (Ps = 0.001), and the CP (µm) decreased from 568.32 ± 32.33 to 521.68 ± 49.98 (*p* = 0.001) (Table 4). Further, in terms of the corneal symmetry, the SUM and DISTANCE decreased postoperatively (from 141.88 ± 48.24 to 66.21 ± 15.22 µm, and from 1.11 ± 1.14 to 0.46 ± 0.40 mm, respectively) (Ps = 0.001), and the kappa angle (degree) decreased postoperatively (from 4.58 ± 1.19 to 2.42 ± 1.75) (*p* = 0.02) (Table 4, Figure 1 and Figure 3). The TBUT (second) markedly increased from 8.51 ± 2.97 to 19.39 ± 3.69 (*p* = 0.001), but the tear osmolarity (Osm/L) decreased from 0.55 ± 0.15 to 0.23 ± 0.12 (*p* = 0.001) (Table 4).

**Figure 5:**
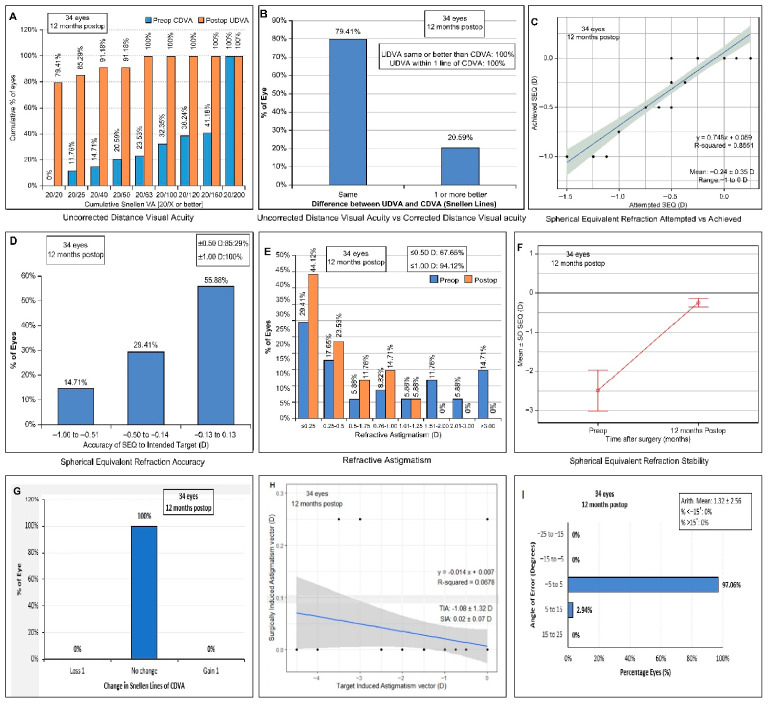
Nine standard graphs for visual and refractive outcomes of all 34 eyes 1 year after L-LAK (LASEK–laser asymmetric keratectomy). (**A**) Cumulative UDVA versus CDVA. (**B**) Change in Snellen lines from preoperative CDVA to postoperative CDVA. (**C**) Attempted versus achieved spherical equivalent refraction (SEQ)with linear regression and correlation values. (**D**) Accuracy of postoperative SEQ to an emmetropic target. (**E**) Cumulative preoperative and postoperative refractive astigmatism. (**F**) Stability of SEQ demonstrated as the trend in the mean SEQ at the preoperative visit and 12 months postoperation. (**G**) Change in Snellen line of CDVA. (**H**) Target induced astigmatism (TIA) versus surgically induced astigmatism (SIA). (**I**) Histogram of refractive astigmatism angle of error.

Standard graphs summarizing the refractive outcomes are shown in Figure 5. As can be seen, 79.4% (27/34) of the eyes had a UCVA of 20/20 or better (Figure 5A); 100% of the eyes had no changes in the CDVA; in 79.4% of the eyes, the UDVA and CDVA stayed the same; 20.6% of the eyes had a CDVA better than their UDVA (Figure 5B); 55.9% of the eyes had plano refraction; and 29.4% of the eyes had an SE within 0.50 D of the intended value (Figure 5C,D). At 1 year postoperation, 44.1% (12/34) had ≤0.25 D of residual astigmatism, 23.5% had ≤0.50 D of residual astigmatism (Figure 5E), and 97.1% of the eyes had a refractive astigmatism angle of error within 5 degrees (Figure 5I). The SE had a stability of −0.40 ± 0.43 D (Figure 5F), no changes in the Snellen line of the CDVA were seen (Figure 5G), and the surgically induced astigmatism vector decreased (Figure 5H). No postoperative corneal ectasia was seen.

## 4. Discussion

This study showed focal corneal steepening over +47 D and the thinnest pachymetry value was 568.32 ± 32.33 μm, exceeding the criterion of 500 μm for keratoconus suspect. But the most important finding was the SUM and DISTANCE: the greater the SUM and DISTANCE, the more that postoperative biomechanical effects caused optical aberrations. The SUM was 141.88 ± 48.24 μm before operation, but after operation, it decreased to 66.21 ± 15.22 µm, meaning that the cornea was symmetric. Also, the DISTANCE decreased from 1.11 ± 1.14 mm preoperatively to 0.46 ± 0.40 mm postoperatively. The Orbscan maps were used because this enabled easy calculation of the DISTANCE and SUM for evaluating corneal symmetry.

KCS is absolutely avoidable for laser refractive surgery because of postoperative complications [19,20,21,22,23,24,25,26,27,28,29]. But in this study, C-LAK showed good 1-year postoperative outcomes without corneal ectasia and focal corneal steepening, because it could ablate refractive errors and also make the cornea symmetric with selective ablation of the thicker peripheral cornea (crescentic customized ablation). Also, the postoperative corneal symmetry, evaluated using the SUM and DISTANCE shown on an Orbscan map, increased markedly, and the focal steep keratometric curvature markedly flattened postoperatively, showing good visual results without postoperative corneal ectasia and focal corneal steepening.

When the SUM is >80 µm, biomechanical interaction between the corneal thickness, corneal stiffness, and IOP causes the steepening of thinner regions postoperation, leading to corneal ectasia [13,14,15,16,17,18,22,23,24,25,26,27,28,29]. Postoperative corneal symmetry was one of the key findings for maintaining good outcomes without postoperative corneal ectasia [13,14,15,16,17,18].

So, in this study, we mainly compared the preoperative and 1-year postoperative focal steepening, keratometric curvature (Kmax), and peripheral corneal thickness asymmetry (SUM and DISTANCE). The keratometric curvature (Kmax) decreased from +48.21 ± 0.89 preoperatively to +44.47 ± 1.38 postoperatively. The SUM and DISTANCE were markedly decreased postoperatively and are important parameters for evaluating outcomes [13,14,15,16,17,18]. In this study, the SUM and DISTANCE were measured with Orbscan maps. The corneal topography was used because it enabled easy calculation of the DISTANCE and SUM for evaluating corneal symmetry [13,14,15,16,17,18]. But for detecting KC stage 1, Scheimpflug topography and anterior corneal OCT are currently used as indicators [10,11,12]. But until now, KCS has not been defined.

With selective, customized, biomechanical ablation of thick cornea using Vision-Up software (Well C, Busan, Republic of Korea) [13,14,15,16,17,18,32,33], C-LAK could be possible in patients with SUM > 80 µm without postoperative complications [14,15,18,32,33]. C-LAK could make the cornea symmetric in patients with KCS [32,33].

It is difficult to compare the visual outcomes from this study with those of other studies because the inclusion/exclusion criteria and testing methodologies are different. Additionally, in this study, all cases had asymmetric corneas and were in the earlier stage of KC, so the main goal of treatment was to correct the corneal morphology to make it symmetric; another aspect was the patients’ age. Those that were middle aged and over 40 years old needed residual myopia causing near-sightedness to be corrected.

In this study, the postoperative Kmax was flattened and also TBUTs (seconds) were markedly increased over 15 s. Most of the patients did not complain of dry eye or blurring postoperatively. It is expected that an uneven corneal curvature due to a greater focal curvature before operation could make the tear film break easily due to tears flowing down, but an even corneal curvature created by flattening the steep cornea postoperatively keeps the tear layer intact and stabilized [32,33,34,35,36]. So, an uneven corneal curvature due to peripheral asymmetric corneal thickness maybe have a correlation with dry eye, but further research will be needed to confirm this.

Corneal remodeling using LAK increases the corneal symmetry by decreasing the SUM to avoid postoperative corneal changes [13,14,15]. L-LAK can enable the ablation of thicker areas of the cornea and correct refractive errors simultaneously compared to conventional laser refractive surgery only, which symmetrically ablates the cornea to correct the refractive errors centrally, so this technique results in good outcomes for patients with KCS, especially with focal corneal steepening over 47 D, without postoperative complications. But the potential risk of postoperative corneal ectasia requires further observation over a longer follow-up period. Additionally, corneal remodeling with LAK could be used (1) to avoid laser refractive surgery’s adverse effects in patients with a SUM ≥ 80 µm [13,14,15,18], (2) as a novel enhancement in patients with myopic regression after laser refractive surgery [16,17], and (3) to manage the postoperative corneal adverse effects of cataracts or glaucoma operations [17,36]. In the future, in cataract surgery on eyes with a poor corneal shape, simultaneously performing Femto-LAK (LAK performed using a Femtosecond laser) will be necessary to improve optical aberrations by corneal remodeling. Lastly, it could be used as (4) a new treatment in KC stage 1 or clinical KC with peripheral asymmetry of the corneal thickness [28,29,32,33]. Further research on LAK as a customizable method will be needed.

Regarding astigmatism correction, regular astigmatism was corrected with original ablation of the refractive errors, the same as with conventional laser refractive surgery such as LASIK etc., but irregular corneal astigmatism was resolved after corneal remodeling using LAK.

This study has several limitations that should be considered when interpreting the results: First, it was a single-center retrospective study; therefore, there may have been some selection bias. Second, the sample size was relatively small, and there was a relatively short follow-up period of 1 year. Third, it was not a comparative study between C-LAK and conventional laser refractive surgery in KCS. Fourth, there was no information gathered on HOA or biomechanical data. Finally, only Korean patients were included; thus, the findings may not be generalizable to other ethnic groups.

As a result, C-LAK decreased the SUM and DISTANCE and increased the corneal symmetry; there were no postoperative complications; and the TBUT increased with good results in terms of visual outcomes in myopic patients with keratoconus suspect.

## Figures and Tables

**Table 1:** Demographic data of patients (mean ± SD).

Item	N = 20 pts (34 Eyes)
Age (year)	41.0 ± 13.5
Males–Females	7:13
Both–One Eye Operated On	14:6

Pts = patients.

**Table 2:** Intra-operative findings (mean ± SD).

Item	N = 20 pts (34 Eyes)
Optic zone (mm)	6.35 ± 0.09
Ablation depth (µm)	64.53 ± 24.41
Myopic shift (diopters) due to LAK	−1.92 ± 0.48
Residual stromal depth (µm)	444.26 ± 36.20

LAK = laser asymmetric keratectomy; pts = patients.

**Table 3:** Comparative findings between preoperative and 1-year postoperative status.

Findings	Preoperation	1-Year Postoperation	*p*-Value
SE (D)	−2.57 ± 1.04	−0.40 ± 0.43	0.001
Sphere (D)	−2.12 ± 4.31	−0.21 ± 0.43	0.001
Cylinder (D)	−1.02 ± 2.27	−0.45 ± 0.42	0.003
UDVA (LogMAR)	0.81 ± 0.31	0.07 ± 0.16	0.001
IOP (mmHg)	15.85 ± 2.24	14.94 ± 1.30	0.057
Pupil size (mm)	4.09 ± 0.71	3.91 ± 0.64	0.059

SE = spherical equivalent; D = dioters; UDVA = uncorrected distance visual acuity; LogMAR = logarithm of minimum angle of resolution; IOP = intraocular pressure.

**Table 4:** Comparative findings between preoperative and 1-year postoperative corneal status.

Findings	Preoperation	1-Year Postoperation	*p*-Value
Keratometry			
Kmean	45.23 ± 0.93	42.67 ± 1.84	0.001
Kmax	48.21 ± 0.89	44.47 ± 1.38	0.001
Pachymetry			
CP (µm)	568.32 ± 32.33	521.68 ± 49.98	0.001
Corneal symmetry			
SUM (µm)	141.88 ± 48.24	66.21 ± 15.22	0.001
DISTANCE (mm)	1.11 ± 1.14	0.46 ± 0.40	0.001
Kappa angle (degree)	4.56 ± 1.19	2.42 ± 1.74	0.02
Tear film			
TBUT (second)	8.51 ± 2.97	19.39 ± 3.69	0.001
Tear osmolarity	0.55 ± 0.15	0.23 ± 0.12	0.001

CCP = central corneal power; D = diopters; BFS = best-fit-sphere; CP = central pachymetry; SUM = sum of deviations in corneal thickness in four directions on Orbscan map; DISTANCE = distance between the maximum posterior elevation (best-fit-sphere [BFS]) and the visual axis; D = diopters, TBUT = tear break-out time.

## Data Availability

The datasets generated and/or analyzed during the current study are not publicly available due to privacy and ethical reasons but are available from the corresponding author on reasonable request.

## Abbreviations

The following abbreviations are used in this manuscript:

BFS	best-fit-sphere
CP	central pachymetry
FDA	Food and Drug Administration
FFKC	forme fruste keratoconus
IOP	intraocular pressure
KC	keratoconus
KCS	keratoconus suspect
LASEK	laser epithelial keratomileusis
L-LAK	laser asymmetric keratectomy
PRK	photorefractive keratectomy
SE	spherical equivalent
